# A Long-Term Terrestrial Laser Scanning Measurement Station to Continuously Monitor Structural and Phenological Dynamics of Boreal Forest Canopy

**DOI:** 10.3389/fpls.2020.606752

**Published:** 2021-01-07

**Authors:** Mariana Batista Campos, Paula Litkey, Yunsheng Wang, Yuwei Chen, Heikki Hyyti, Juha Hyyppä, Eetu Puttonen

**Affiliations:** Department of Remote Sensing and Photogrammetry, Finnish Geospatial Research Institute (FGI), National Land Survey of Finland, Masala, Finland

**Keywords:** TLS time series, vegetation phenology, forest dynamics, boreal forest monitoring, circadian movement

## Abstract

The terrestrial laser scanner (TLS) has become standard technology for vegetation dynamics monitoring. TLS time series have significant underlying application in investigating structural development and dynamics on a daily and seasonal scale. However, the high potential of TLS for the monitoring of long-term temporal phenomena in fully grown trees with high spatial and temporal resolution has not yet been fully explored. Automated TLS platforms for long-term data collection and monitoring of forest dynamics are rare; and long-term TLS time series data is not yet readily available to potential end-user, such as forestry researchers and plant biologists. This work presents an automated and permanent TLS measurement station that collects high frequency and high spatial resolution TLS time series, aiming to monitor short- and long-term phenological changes at a boreal forestry field station (0.006° angular resolution, one scan per hour). The measurement station is the first of its kind considering the scope, accuracy, and length of the time series it produces. The TLS measurement station provides a unique dataset to monitor the 3D physical structure of a boreal forest, enabling new insights into forest dynamics. For instance, the information collected by the TLS station can be used to accurately detect structural changes in tree crowns surrounding the station. These changes and their timing can be linked with the phenological state of plants, such as the start of leaf-out during spring growing season. As the first results of this novel station, we present time series data products collected with the station and what detailed information it provides about the phenological changes in the test site during the leaf sprout in spring.

## Introduction

Vegetation dynamics can be monitored through non-destructive methods, using close-range remote sensors, such as terrestrial laser scanning (TLS). The high penetrability of the laser beam associated with the ability to acquire multiple returns per transmitted pulse (Wehr and Lohr, 1999) makes laser scanning a unique measurement tool for data acquisition (Hyyppä et al., 2008) and the three-dimensional representation and biophysical analysis of forest environments (Eitel et al., 2016). TLS can automatically acquire 3D measurement of trees with high-frequency (minute level) and high-spatial accuracy (centimeter-level). TLS scans can be acquired from different perspectives close to ground and regardless of external lighting conditions. The standard TLS equipment uses low-energy infra-red laser light that has high reflectance from vegetation. These features allow constant extraction of forest structural parameters at an individual tree level without interfering with their internal processes.

The potential of TLS data to estimate forest structure parameters for ecological and economic forest analyses has been extensively presented in earlier literature, where the main focus has been usually in estimating the diameter of the breast height (DBH, Liang et al., 2012; Pueschel et al., 2013), leaf area index (Béland et al., 2011; Kargar et al., 2019), tree height (Maas et al., 2008) and aboveground biomass (AGB) (Olsoy et al., 2014). However, according to Newnham et al. (2015), TLS technology not only provides forest structural parameters, but also a whole plot-level forest structure together with dynamics at an unprecedented level of detail, which is still underexplored. The feasibility of terrestrial LiDAR data to detect and monitor long-term vegetation dynamics in forest and crop areas have been discussed in previous works, including plant growth (Yu et al., 2004, 2006; Olivier et al., 2017; Guo et al., 2019), DBH increase (Liang et al., 2012), AGB change (Kaasalainen et al., 2014; Srinivasan et al., 2014) and spring sprouting and flowering (Olsoy et al., 2014; Calders et al., 2015). Recently, new studies have also used TLS data for monitoring short-term phenomena, such as circadian rhythms and foliar nyctinasty in different plants and tree species (Puttonen et al., 2015, 2016, 2019; Zlinszky et al., 2017; Herrero-Huerta et al., 2018; Bakay and Moravčík, 2020). These studies highlight the high potential of setting up a permanent TLS measurement station as a new non-destructive tool for boreal forest dynamics monitoring.

Monitoring seasonal vegetation phenomena with TLS requires long-term time series with scan repetitions at monthly or annual intervals. Long-term vegetation monitoring provides the timing of recurring seasonal vegetation dynamics (phenology) and plant phenotype during growth and development, which have strong interactions with Earth’s climate–biosphere system and global environmental changes (White et al., 2009). For instance, systematic shifts at phenophase transition dates, such as start of leaf-out at the beginning of the growing season in spring in boreal forests, are a key phenological indicator of the effect of climate change (Calders et al., 2015). Therefore, improvements in the measurement accuracy of regional to global scale vegetation dynamics can improve our understanding of inter-annual variability in terrestrial ecosystems, the effects of climate change and climate–biosphere interactions. The majority of existing works that have investigated long-term vegetation dynamics have been performed at an individual tree level within a limited time period (Kaasalainen et al., 2010, 2014; Kankare et al., 2013).

Kaasalainen et al. (2014) show in a set of laboratorial and controlled field experiments that TLS time-series measurements combined with 3D quantitative structure modeling can detect and quantify biomass changes in individual trees. Biomass changes in a single maple tree in the field monitoring experiment were detected in a five-scan time series acquired between February 2011 and November 2013 in Finland. In this work, continuous plot-based monitoring with TLS was suggested as a future direction to study biomass change dynamics. Liang et al. (2012) performed two TLS data acquisition using a Leica HDS6000 terrestrial laser scanner (Leica, [Heerbrugg], Switzerland) of a boreal forest plot at Evo, Finland. The first field campaign was done in March 2008 and subsequently in August of the same year. Bi-temporal changes were detected using the voxelization method. The point density in each voxel element at different times was compared to detect the tree changes, in which a point is defined in this paper as a 3D coordinate (*X*-, *Y*-, and *Z*-) measured by TLS principals. Changes in the DBH were estimated with centimetric accuracy (∼1 cm). They also reported canopy density change due to seasonal growth. Further studies focused on investigating volume and biomass changes over time. For example, Srinivasan et al. (2014) and Olivier et al. (2017) proposed new methods to quantify biomass changes over time base on TLS time-series. Srinivasan et al. (2014) performed multi-temporal single scans using Leica ScanStation2 (Leica, [Heerbrugg], Switzerland) on three plots in November 2009 and November 2012 at a Loblolly pine forest (Huntsville, United States), aiming to statistically estimate tree-level AGB. Olivier et al. (2017) studied crown dynamics and architectural developments in sugar maple and coniferous trees species in Quebec, Canada using TLS time-series obtained in 2013 and 2015. Finally, earlier literature has also shown the use of TLS in studying spring phenological changes, such as Olsoy et al. (2014) and Calders et al. (2015). Olsoy et al. (2014) scanned three study sites of sagebrush plants in the Great Basin (United States), during the spring (May 2012) and the fall season (October 2012) to further test the ability of the TLS to quantify seasonal changes in green biomass. Statistical models of shrub canopy volume were derived from TLS point clouds to enable accurate detection of seasonal differences in green biomass. Calders et al. (2015) performed a long-term TLS time-series to detect changes in leaf area index over time in a deciduous forest in the Netherlands. The TLS measurements were performed between February and June 2014. The scan frequency varied between three (February to April), two (May) or one (June) measurements per week (total of 48 scans), according to the expected changes in phenology during this period. The TLS time series clearly show the green season.

Over the last 5 years, new studies have detected and quantified circadian movements in the tree branches and foliage of full-grown trees using TLS data acquired with short timescale measurements that have had an hourly or even more frequent scanning repetition rate (Puttonen et al., 2015, 2016, 2019; Zlinszky et al., 2017; Herrero-Huerta et al., 2018; Bakay and Moravčík, 2020). Puttonen et al. (2016) used TLS time series acquired in two geographically different locations (Finland and Austria) to detect short-term vegetation dynamics. The scanning interval was approximately 1 h in Finland and 10 min in Austria, enabling the detection of a vertical movement between 5 and 10 cm. Subsequently, Zlinszky et al. (2017) performed an 18-scan TLS time series, aiming to measure and differentiate nocturnal changes in 22 different plant species over a 12-h period with repeated scan acquisitions. They reported about different types of circadian movements in the studied species with the highest movement amplitude around 2 cm. Herrero-Huerta et al. (2018) detected leaf displacements and volume variation of two *Calathea roseopicta* plants in a controlled environment experiment in which temperature, relative humidity and light conditions were measured continuously. The measurements were taken in an indoor environment over 2 days. Puttonen et al. (2019) reported a new method to monitor circadian rhythms from point cloud time series. They studied overnight movements in two Norway maples from sunset to sunrise with 20-min scan repetitions that resulted in 130 scans in total. Circadian movements up to 10 cm were identified on branch tips. Recently, Bakay and Moravčík (2020) monitored branch and leaf displacement in lime trees under a controlled environment using high precision 24-h TLS time series. These authors proposed a new method of movement quantification of monitored saplings focused on target point monitoring.

Even though TLS has already shown its potential in vegetation structure mapping and dynamics monitoring, fully automated TLS platforms for long-term data collection and monitoring of forest dynamics are rare. Several reasons exist for this: high-performance TLS scanners are still expensive to date and often repeated high-resolution scans produce large datasets whose processing, transfer and storage present a challenge of their own. Moreover, the use of high-frequency TLS time series in plant dynamics monitoring is a new research topic, where the best practices and methodology are being developed. These limitations present a major bottleneck in the quantitative analysis of plant phenology with TLS time series data. Thus, the full potential of long-term TLS time series data in increasing knowledge is not yet readily available in other disciplines like plant biology, forestry, and forest ecology.

This technical study aims to narrow the gap in utilizing TLS time series data. We present here a pilot system of a permanent TLS measurement station that monitors a boreal forest site with high spatial and temporal resolution. The station monitors temporal phenomena in the surrounding vegetation. The platform is located in a forest research station (Hyytiälä, Finland). To the best of the authors’ knowledge, the station is the first of its kind considering its permanent installation and the aimed spatial and temporal resolutions. The main information acquired with the TLS station is accurately enough to enable structural changes detection of different time scales in surrounding tree crowns. The changes and their timing can be linked with the phenological state in plants and their internal processes that drive the changes. The plant state information is available from the already existing sensor from SMEAR II (Hari et al., 2013). The study gives a detailed description of the TLS system construction, data acquisition routines, data transfer and storage solutions, and preprocessing steps required to produce structural change information of individual trees on different time scales. Different data product examples are introduced to determine the start of spring growth in a few monitored test trees. We conclude the study by discussing the potential of a permanent TLS measurement station in providing both short and long term time-series data to better analyze and understand phenology and dynamics in forests.

## TLS Measurement Station

The TLS measurement station development was performed with practical questions such as the test area and installation location, available hardware options and system configuration in mind. This section describes our solutions to these three practical questions to set up the automated operational measurement station.

### The Test Area and the Location of the System

**FIGURE 1:**
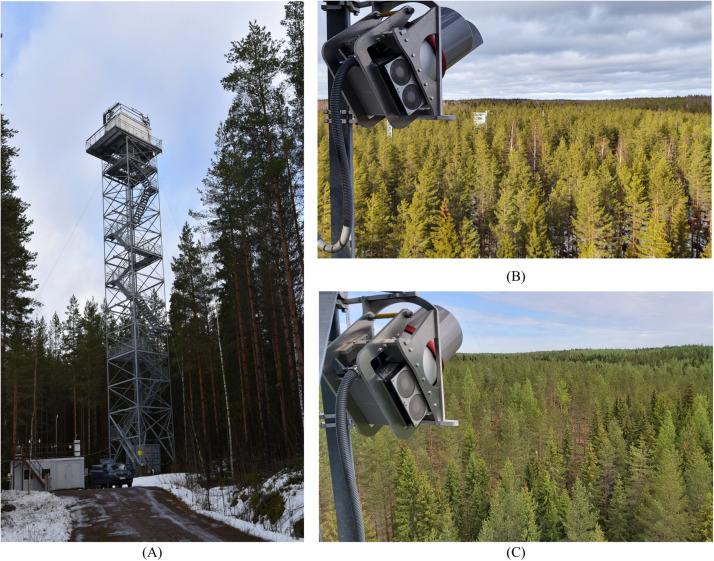
**(A)** the 30-m high measurement tower. The laser scanner is installed in a specially manufactured aluminum frame in the rear corner of the tower facing the test forest. **(B)** The winter view over the test forest next to the RIEGL VZ-2000i laser scanner in February 2020. **(C)** The summer view over the test forest in June 2020. The thick cable coming from the protective hood houses the data and power cables between the scanner, its external power unit and the measurement computer (not in figure).

The TLS measurement station is installed in the Hyytiälä research forest (61°51′N, 24°17′E). The research site has a 58-year-old (2020) test forest located in the Hyytiälä forestry field station^1^ which was established as a practice area for forestry studies in 1910 by the Finnish Government and is currently administrated by the Faculty of Agriculture and Forestry at the University of Helsinki. The forest has been constantly monitored since 1995 by the station (SMEAR II) to study the relationship between the ecosystem and the atmosphere (Hari et al., 2013). The SMEAR II station provides data analyses, such as forest ecophysiology and productivity, soil and water balance, meteorology, solar and terrestrial radiation, and atmospheric aerosols^2^. These resources enable the implementation of new high-quality research activities with different sensor systems, which was the main motivation to install the permanent TLS measurement station at Hyytiälä (Figure 1). The TLS measurement station (scanner and local server) was installed near to the top of a 35-meter high tower (Figure 1A), where a laboratory is built in a standard cargo container. From this point of view, the scanner can monitor an area of approximately 263 m × 169 m. At least 400 trees had stem and canopy clear visible from the tower point of view. The scanned forest area is a mixed forest dominated by coniferous trees typically found in the boreal forests of Finland. The three main tree species in the test area are the coniferous Scots pine (*Pinus sylvestris*) and Norway spruce (*Picea abies*) mixed with deciduous Silver birch (*Betula pendula*), which are also the dominant tree species in Finnish forests. Tree biometric parameters (e.g., tree height, DBH, stem surface area, forest density and stand basal) of these dominant trees at the test area can be found at and Machacova et al. (2019) and Chen et al. (2020). Figure 1A shows the tower where the measurement station was installed about 30 m above ground. Figures 1B,C give an overview of the surrounding test forest as viewed next to the laser scanner facing the forest.

### The Hardware and Setup

The capabilities of both the TLS equipment and computational capacity to process and store TLS data have improved significantly in recent years. There are a wide variety of TLS systems available on the market with different prices and technical properties. The increasing availability of equipment has opened many possibilities to tailor the system configuration according to the application and accuracy requirements. In the present case, the long-term monitoring and analysis of a forest were specified to have sub-centimeter positional and minute-level temporal resolution, which is attainable with accurate three-dimensional point clouds acquired using the state-of-the-art laser scanner. The TLS measurement station consists of a RIEGL VZ-2000i (RIEGL Gmbh, Horn, Austria) laser scanner (Class 1A) installed in a weather-protected hood (RIEGL PHA200/1000) to ensure permanent outdoor measurements even in harsh Finnish winters. The hood is tilted 60 degrees down with a custom-built frame to give a better field of view (FOV) toward the forest, avoiding canopy occlusions. However, tree features that are not facing the scan, such as part of the tree stems, will be naturally occluded.

To capture tree dynamic behavior accurately, the scanning requirements of the scanner were set (a) to be able to spatially resolve two neighboring points at least with 0.01 m spacing at a 100-m range and (b) to be able to scan over the whole FOV with the required point resolution at least twice per hour. The laser distance measurement is performed by the high-precision time-of-flight measurement mode, with a nominal distance accuracy of 3 mm and a beam divergence of 0.27 m at 100 m distance. The angular resolution in vertical and horizontal directions can range from 0.6° up to 0.0017°, considering a scanning mechanism with a vertically rotation multi-facer mirror and a horizontally rotation head with a maximum FOV of 100 and 360 degrees, respectively. The technical properties of the laser scanner are summarized in Table 1. However, these requirements led to high-density data collection and resulted in large data sets that have to be transferred and stored efficiently. To guarantee data storage and transmission, a separate computer was set up at the measurement station to act as a local server for temporary data storage and scheduled daily data transmission. Data acquired by the scanner is stored at the local server and transmitted to a separate network storage in the National Land Survey of Finland (NLS-NAS) network on a daily basis. The daily data is stored at the local server for several days as a temporary backup in case of network breakdowns. More information about the data acquisition and transmission is given in Section “Data Acquisition, Transmission and Storage.”

**TABLE 1:** Technical specifications of the terrestrial laser scanner.

Scanner	RIEGL VZ-2000i
Type/Laser class	Time of Flight/Class 1A
Wavelength	1,550 nm
Scanning mechanism	Vertically rotating multi-facet mirror, horizontally rotating head
Max. FOV (vertical/horizontal)	100/360
Scan frequency	50–1,200 kHz
Max. number of returns per emitted pulse	8
Max detection range (for natural targets with 10% reflectivity)	600 m
Distance measurement accuracy/precision	5 mm/3 mm
Beam divergence (rad)	0.27 mrad/100 m

### The System Configuration

As a pioneer station of this kind, a proper configuration for the point cloud time-series collection was first evaluated in a preliminary study during 2019 (Campos et al., 2020). The measurement station operation and automation concept were first piloted in a set of several long-term monitoring experiments between the beginning of April and the end of October in 2019 in a test area of about 227 m × 143 m in Southern Finland (60°09′N, 24°32′E) at the Finnish Geospatial Institute (Campos et al., 2020). After the pilot experiments, the measurement station was moved to the Hyytiälä forest station in February 2020 where it reached full operability status in March 2020. Based on the experiences from the pilot experiments, the TLS measurement station was configured to measure the surrounding forest with 1,200 kHz frequency up to a detection range of 138 m. The measurement scanning window was set with a FOV of 87° vertically and 151° horizontally. The angular resolution in vertical and horizontal directions was set to 0.006°, which corresponds with the required 0.01 m horizontal and vertical point spacing at a 100-m distance from the scanner. These fixed measurement configurations were selected for collecting the long-term time series from the Hyytiälä test forest site and the targets in it (trees and understory vegetation). The configuration resulted in an individual scan time of 21 min and produced about 600 million points per scan.

## Time Series Data Acquisition and Processing Framework

**FIGURE 2:**
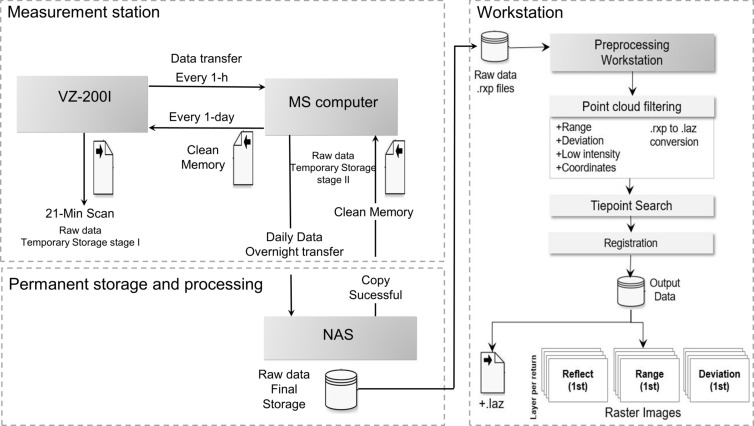
TLS measurement station data acquisition and pre-processing framework. Each block defined with a dashed line represents an independent unit that can be physically separated from the others.

The collected time-series data acquisition and processing framework are illustrated in Figure 2. The schematic diagram summarizes the framework that includes the TLS measurement station data acquisition (see section “Data Acquisition, Transmission and Storage”) and data preprocessing in a separate workstation after data collection (see section “Pre-processing and Output”).

### Data Acquisition, Transmission, and Storage

The time-series data collection workflow is fully automated using Python and shell scripts, including the setting up of the laser scanning configurations, daily project initialization, scan acquisition over a preset time window, local data storage management, daily data transfer from the measurement station to the NLS-NAS and a weekly reboot of the laser scanning system. This section describes in detail how the data acquisition workflow is programmed in the devices embedded in the TLS measurement station.

Every day, a new data collection project is initialized in the TLS measurement station. At present, a configured 21-min scan data collection and local storing in the measurement station are performed every hour, resulting in 24 scans per day (192 GB). The TLS measurement (‘scan’) creates a point cloud file in.rxp data format (proprietary RIEGL) and related cover metadata such as scan final pose and weather parameters. Each individual scan produces about 8 GB of data with the used scanning configuration. The laser scanner is capable of collecting two scans per hour, which would double the temporal resolution of the daily measurements given that all data can be transferred and processed on time. As complementary data, the local weather parameters (wind speed, wind direction, temperature, precipitation and relative humidity) with a 1-min collection interval are scheduled to download automatically from the Helsinki University SMEAR II station. The weather data is associated with each scan day-project. Additional reference data, collected in other on-going research projects in the test area can be added similarly to be used in future analysis and modeling.

Data from each individual scan is then transferred from the scanner and stored temporarily in the local server at the measurement station unit (MS). The scan files (.rxp and metadata) are regularly copied over an SSH connection from the laser scanner to the local server 30 min after a scan has been initiated. The local transfer between the measurement station devices takes around 19 min. Therefore, scanning and backup are performed within 1 h. The copy success is confirmed by comparing the file size on both devices. In case of a copy failure, the copying is performed again the following hour. The laser scanner has an in-built storage disk with 886 GB capacity. The daily workflow regularly takes up to 40% (the present and the previous day data) of the available space leaving the remaining 60% as a temporary data storage to mitigate possible copy failures. Thus, about five full days’ worth of data can be stored in the scanner in total.

Next, the daily project is scheduled to be copied overnight via an SSH connection to the permanent storage in NLS-NAS. In this case, the raw measurement station data is saved in the network storage, which can be accessed from a separate workstation computer also located in the NLS network. However, the NAS can be established in other locations or in cloud storage (e.g., CSC). The overnight transmission takes around 11 h, with the present average transfer speed of 8 MB/s. Until the transmission between the local server and the NLS-NAS is confirmed to be successful, a daily project backup is kept in the MS local server. This prevents data loss in the case of a failure or low data transfer speeds caused by any problem in the network (e.g., energy or network break). The MS local server has a capacity to keep daily project backups up to 12 days. Each transfer routine creates automated email warnings in case of any transfer failures to allow quick maintenance. Finally, after the data copying to the NLS-NAS is confirmed to be successful with file size checking, the original data is deleted from the MS local server.

### Pre-processing and Output

The workstation performs systematic data pre-processing in a separate workflow to produce analysis-ready 3D point cloud and raster image data products, including as output filtered full point cloud (.laz), individual trees point clouds (.laz), and range, reflectance and deviation raster images (.tiff) for each scan.

#### ASPRS LAZ Point Cloud

**FIGURE 3:**
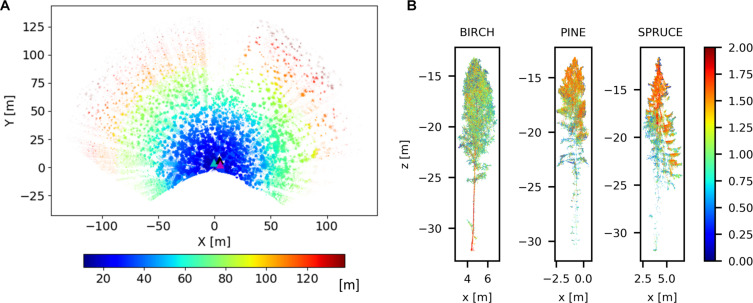
Example of output point clouds from Hyytiälä test area acquired on 12 April 2020. **(A)** Top view of a filtered full point cloud colorized by range. The three example tree positions are highlighted with magenta (birch), black (Spruce), and cyan (Pine) triangles. **(B)** Individual tree points of a birch, a Scots pine and a Norway spruce colorized by point reflectance.

The initial step in creating the output products was the conversion of the point clouds from the data collection format .rxp into a more widely supported open format, the ASPRS LAS. The raw point cloud files were converted to compressed to the ASPRS .laz version 1.4 format that supports additional data fields. The conversion was performed using a C/C++ routine that calls the dynamically linked RIEGL RivLib (version 7.1) and LASzip (version 3.4; RapidLasso Gmbh, Gilching, Germany) libraries (DLLs). After data conversion, the resulting point cloud in .laz format contains the 3D coordinates (*X*-, *Y*-, and *Z*-) of the points in a local reference system, point return number (scalar from 1 to15), number of returns (scalar from 1 to15), intensity (dB ranging from 0 to1), scan angles (theta and phi in degrees), reflectance (dB), return pulse deviation (where a larger value for deviation indicates larger return pulse width), range (m) and the internal scanner coordinates (row and column). The last five parameters were stored as user-defined extra bytes in the .laz content. Range (ρ), scan angles (θ, φ) and the internal scanner coordinates are not standard output values from an .rxp file. Their values were computed within the conversion routine using the 3D coordinates of the point cloud in a local reference system (*X-*, *Y*-, and *Z*-), in which the point cloud origin (0, 0, 0) is set to the laser scanner position. The computed values were associated with each point (subscript *i*) during the .laz file writing. Range values and scan angles can be calculated as presented in Equation 1 and 2, respectively. Figure 3 shows examples of a full and individual tree point clouds. A full point cloud visualization from a top perspective and colorized by range is presented in Figure 3A. Figure 3B shows an example of three individual tree point clouds of the dominant tree species in the Hyytiälä test area (Silver birch, Scots pine and Norway spruce). The tree point clouds are colorized by their reflectance values. The positions of the example tree point clouds is highlighted in Figure 3A with colored triangles.

(1) $$\rho_{i}=\sqrt[{2}]{X_{i}^{2}+Y_{i}^{2}+Z_{i}^{2}}$$

(2) θi=cos−1(Ziρi);φi=tan−1YiXi

#### Rasterization of Point Cloud and Generation of TIFF Images

The scan data were also converted into raster images (TIFF) using the point-wise range, reflectance and return pulse deviation values. The raster image approach provides a significant advantage in developing new analysis methods because existing, well-established, image processing techniques can be readily utilized in data processing and analyses. Image-based analysis methods are also typically faster than point-cloud based methods. Moreover, mapping the point clouds into raster images can be done here without loss of resolution by using the native point parameters of each scanned point in the point cloud, such as point range and reflectance values. Thus, the results of any processing or filtering operation can be transferred directly back to the other representation. Examples of raster image operations applicable in the raster images derived from TLS data include the automatic marker-free registration of the point cloud time series using feature-based matching (FBM) methods (Houshiar et al., 2015; Urban and Weinmann, 2015), and object detection and segmentation (Bienert and Schneider, 2013). Stable features presented in the test area, such as fixed construction containers around the tower, can be monitored over the image time-series to detect and correct scanner leveling problems, which can affect the time-series data analyses.

**FIGURE 4:**
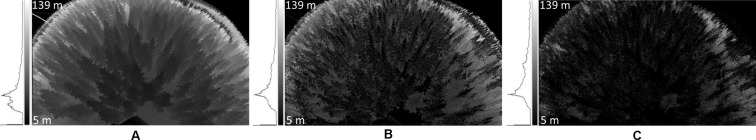
Example of output raster images colorized in gray scale byrange from Hyytiälä test area acquired on May 11, 2020, in which **(A–C)** show the image layers from first to the third return. The histograms on the left side of each subfigure show the range distribution of detected points.

With this motivation, the point clouds acquired in each scan were converted into a four-channel raster image. Each imaging channel corresponded with the return number of the returning laser pulses. Only the first four laser return pulses were considered as they accounted for 99.9% of all returns. The main steps in the algorithm to generate raster images are the definition of image projection, the definition of raster image frame and determination of color values (Vosselman and Klein, 2010). These steps were implemented with Python scripts in the processing workflow. Regarding image projection, the raster image was generated considering an equirectangular projection. The equirectangular projection is one of the most straightforward projections for mapping the 3D point cloud to the image plane, since the scan angles can be directly associated to the image coordinates (Houshiar et al., 2015). Therefore, the image coordinates, i.e., the row and column values of each point that composes the 3D point cloud were computed based on the scan angles (θ and ϕ), the angular resolution (α = 0.006°), and the measurement scanning window with a horizontal and vertical field of view of 87° and 151°, respectively. The equirectangular projection supports 360° (2π) in the horizontal field of view and 180° (π) in the vertical field of view. Therefore, row and column values (c, r) were normalized per π to fix the image frame size (n_C_ × n_R_). The projection resulted in raster images with dimensions of 8,013 and 4,618 pixels with a pixel size of 0.0019 degrees (0.35 mm). The transformation equations of this projection are presented in Equation 3 (Bienert and Schneider, 2013). An example of three raster image layers of a range image from the first to the third return can be found in Figures 4A–C, respectively. The gray-scale values were set to cover range values between 5 and 139 m from the scanner. We can notice in the images that the deciduous trees, such as Silver birch, in the test site provide more multiple returns than coniferous trees.

(3) $$r_{i}=\frac{\theta_{i}-\min\left(\theta \right)}{\alpha };c_{i}=\frac{\varphi_{i}-\min\left(\varphi \right)}{\alpha }$$

## Examples of TLS Measurement Data Application

This section presents two examples of vegetation dynamics monitoring using the TLS measurement station output. The examples demonstrate the achievable level of detail with long-term TLS time series and the potential they can offer for end-users. In the first example, long-term vegetation dynamics are monitored during the spring growth when the first leaves and flowers start to sprout. The start of growth is determined from DBH increase and volumetric change in AGB in an individual tree. The second example illustrates the use of TLS time series data in short term phenomena monitoring, for instance in detecting the circadian rhythms in plant canopy. For both examples, a Silver birch was selected for analysis. Birch wood has high value and usability, which make birch one of the most important commercial species in Finland (Koski and Rousi, 2005). Due to this importance, many studies have been performed since the 1940s to monitor the growth and development of birch stands. An overview about silver birch features and related researches can be found at Heräjärvi (2002). The birch was located about 6 m away from the tower. This tree was 19 m tall and had a DBH of 173 mm. The DBH was manually determined as an average of four caliper measurements in June 2020 (181, 166, 170, and 175 mm) with a standard deviation of 5 mm between the measurements. The position of the Silver birch in the study area is presented in Figure 3. Silver birch is a deciduous hardwood species, easily recognizable due to the light or slightly yellowish wood color. The Silver birch was delineated from the full point cloud dataset during the rxp to .laz conversion. Range, scan angles, and coordinates values were used as delineation thresholds. The bounding box used in delineation was manually optimized and obtained using Cloud Compare software (Cloud Compare, v 2.11, 2020). Example data is available as Supplementary Material. Python (las2np) and MATLAB (las2mat) tools developed to read and write .laz data will be freely available at Gitlab.

### Long-Term Monitoring

After becoming operational, the measurement station has been monitoring the Hyytiälä forest site over the growing season between April and July 2020. The monitoring period consists of 120 daily projects, resulting in a large dataset with about 3 thousand scans and a total size of 23 TB of data. From this dataset, a set of 4 days was selected considering the typical start of leaf growth in the spring and the local weather conditions. The selected dates ranged from early-, middle-, late- spring, and early summer being the May 01, 16, and 30 and June 15. A single scan acquired at the same time of day (1:00 A.M.) from each date is considered here in the long-term analysis. The main criterion for a scan acquisition to be included was to have similar weather conditions with the other selected scans, i.e., wind speeds smaller than 3 m/s (defined as calm and light breeze in Beaufort scale rates), and no precipitation intensities (0 mm/h) at the time of their collection. For instance, about 2,880 scans were collected from April to July 2020. From this, the total of 1,236 scans meet this weather criterion (43%), being 222 scan in April, 316 scan in May, 423 scan in June and 275 scan in July. However, the weather criterion needs to be assessed and selected according to the user application.

#### Volumetric Dynamics on Crown

**FIGURE 5:**
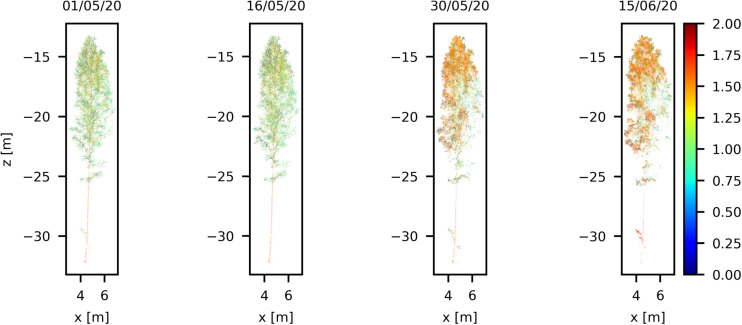
Seasonal variation in a Silver birch crown during the spring growth season visualized with four scans between May and June in 2020. The color scale presents individual laser point reflectance in logarithmic scale (0–2.0).

**FIGURE 6:**
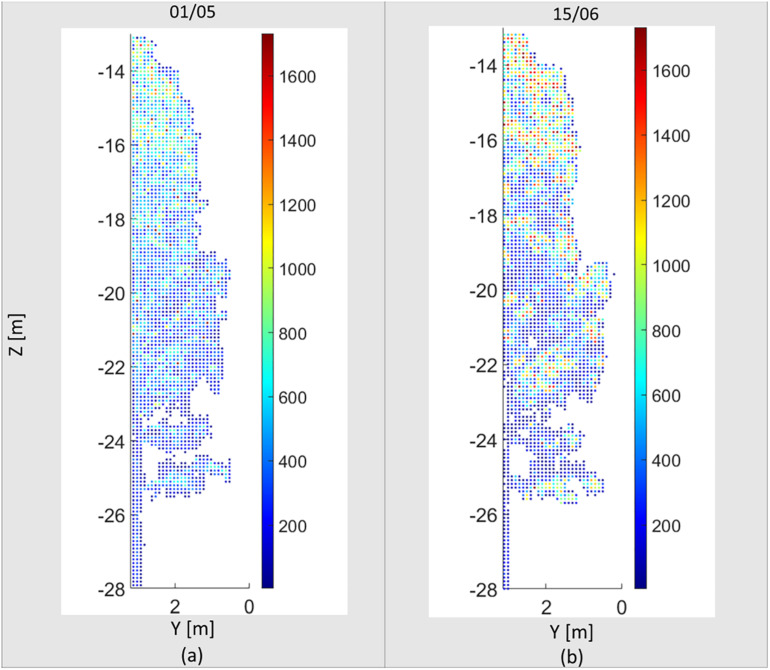
Seasonal variation in a Silver birch crown in the voxel space between May 01 **(a)** and June 15 **(b)**. The color scale presents the number of points per voxels with 0.10 m dimension.

During the monitoring period between early April and middle of June, the air temperature increased from −2 to 10 degrees Celsius after sunset. According to Ruosteenoja et al. (2011), the growing season starts once the mean daily temperature exceeds 5°C, which usually takes place in southern Finland in early May about a month and half after the March equinox. Seasonal phenology changes are clearly visible in the four point clouds selected from the time series. The four point clouds are presented in Figure 5. These point clouds data can be found in the Supplementary Material. The point clouds were colorized with respect to the reflectance parameter values, ranging from 0 to 2. The sprouting of leaves can be visually inspected after the middle of May. Changes in the overall reflectance response in the birch crown can be associated to the sprout of new green leaves during the grown season. The increase in the point cloud density and volumetric change also confirm the leaf sprout and green biomass changes in the birch during this period. For instance, the number of points scanned within the delineated birch bounding box was around 5 million points (4,761,313 points) in early May, increasing by 28.5% to about 6 million points in middle June (6,120,147 points). The changes are also detected by computing the average points in the voxel space (Figure 6). A voxel of 0.10 m was used. The nearest points to the laser scan (half of the tree) was considered. The number of voxels computed was 24,643 and 25,860 for May and June, respectively. The average density of points increased from 137 points per voxel in early May to 157 points per voxel in Middle June (14%). Figure shows the increasing of the point density per voxel, mainly in the tree canopy.

**FIGURE 7:**
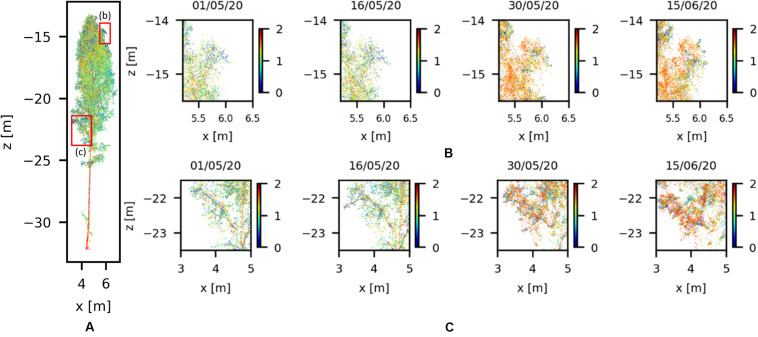
Zoomed in the visualization of Silver birch branches with their point reflectance colored logarithmically (0–2.0) and their weekly variation during spring 2020. Panel **(A)** shows the positions of the branches in the Silver birch tree marked with red boxes, and Panels **(B,C)** show the leaf sprout in the highlighted branches.

In order to demonstrate the leaf sprout with better detail, two branches were selected from of Silver birch tree (Figure 7). The positions of the branches in the Silver birch tree are highlighted in Figure 7A. The differences between the point clouds in the monitoring period show that the leaf sprouting starts between 16 and 30 May. This result can be verified from the meteorological data available at the forest site. According to the SMEAR data from Hyytiälä station, the daily average temperature exceeds 5°C and starts to increase relatively constantly after May19. Therefore, we can conclude that the acquired TLS time-series detects the timing of phenological changes in birch crown accurately.

Accurate identification of phenological indicators – e.g., the start of growing season – is important in applying them in phenological models to understand the growth dynamics (Ruosteenoja et al., 2011). Additionally, the long-term TLS monitoring of a controlled forest area, such as Hyytiälä, will provide annual TLS time-series, enabling novel information to connect TLS data with biological cycles and environmental conditions. Earlier literature (Euskirchen et al., 2006; Calders et al., 2015) discusses the importance of detecting any systematic or temporal shifts in the beginning of the growing season in spring. According to them, the shifts are a key phenological indicator in monitoring the effect of climate change in boreal forests and cultivated areas. The shifts can affect productivity, breeding cycle, and the ability of capture carbon dioxide (CO_2_) from the atmosphere in boreal forests. Changes in productivity will influence the ability of these ecosystems to sequester atmospheric CO_2_.

#### Changes in Stem Diameter

**FIGURE 8:**
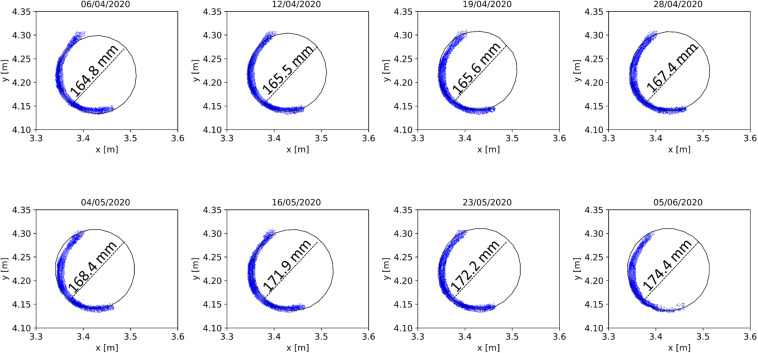
Detection of DBH growth trend from the Silver birch tree point clouds acquired between April 06 and June 05.

Another example of long-term TLS time-series application is the monitoring and estimation of forest structure parameters, such as DBH, tree height and AGB. These parameters are used in a large variety of ecological and economic forest analyses. As discussed by Liang et al. (2012), the detection and quantification of stem changes caused by natural forces and timber harvesting is an important input for many studies on-going in forest management and biomass estimation. Based on our initial analysis, at least 150 tree stems are visible to the scanner in the test area point clouds, considering a normalized height ranging between 1 and 4 m from the ground. This enables the automatic monitoring and determination of DBH over the site. Here, we provide an example of monitoring DBH increase automatically with the TLS time series. The same Silver birch tree as in the previous example was monitored between April 04 and June 05. The results are illustrated in Figure 8.

First, the stem was manually segmented from the Silver birch tree point cloud at 1.3 m height above the ground (±30 cm around). A cylinder surface was then fitted to the set of laser points belonging to the segmented tree stem using the random sample consensus (RANSAC) method (Fischler and Bolles, 1981). This process was equally performed for the data acquired eight different dates from April to June (Figure 8). The segmentation and cylinder fitting were performed with Cloud Compare. The resulting cylinder radii fit to the tree stem segments were 82.4 mm (σ = 3.4 mm), 82.7 mm (σ = 3.6 mm), 83.4 mm (σ = 4.4 mm), 83.7 mm (σ = 5 mm), 84.2 mm (σ = 4.2 mm), 85.6 mm (σ = 3.6 mm), 86.1 mm (σ = 4.6 mm), and 87.2 mm (σ = 3.7 mm), corresponding with estimated DBHs of 164.8 mm (April 6th), 165.5 mm (April 12th), 165.6 mm (April 19th), 167.4 mm (April 28th), 168.4 (May 4th), 171.9 (May 16th), 172.2 (May 23th), and 174.4 mm (June 5th). The reference DBH value of the Silver birch tree was 173 mm (±5 mm), which is the average value of four consecutive manual measurements performed using a caliper taken during a field campaign on June 9th. In this example, the DBH estimated in the begging of June (June 5th) has a discrepancy of 1.4 mm with respect to the reference obtained on June 9th, which is consistent with the standard deviation estimated by Cloud Compare software.

It is important to highlight that this example was performed with only one manually delineated stem segment considering a single tree species (Birch). Thus, further studies are needed to determine the accuracy of the multi-temporal TLS measurements from the measurement station to obtain forest structure parameters in growing trees and to correlate the result with DBH changes. A challenge that can be mentioned is stem occlusions around 1.3 m height due to understory vegetation and canopy growing. Differences between tree species structures needs to be considered in DBH automatic detection. For instance, DBH measurements in coniferous trees, such as Norway spruce, tend to be more challenge due to leaf distribution and possible stem occlusions, especially during the grown season. Therefore, more stems need to be considered in future works for further conclusions. In general, DBH can be estimated in a fully automatic process with an accuracy of around 1 cm on plot level (Liang et al., 2012). Therefore, we believe that the TLS measurement station will provide stable time series to detect DBH variation and growth trends. The DBH estimates from the measurement station data can be correlated in future with ground reference data provided by other sensor systems, like point dendrometers installed in the test area. Combining different time series data will provide input for several new studies focusing on method automation to estimate DBH estimation and detect tree stem changes, among others.

### Short-Term Monitoring

The circadian rhythm can be understood as a near-24-h biological cycle that occurs due to a combination of a plant’s internal physiology and its environmental factors (Sadava et al., 2009). The rhythm can lead to the endogenous movement of leaves and branches that are generated without the influence of external factors such as wind. The circadian movements usually persist under constant environmental conditions, like in stabilized lighting and temperature. TLS data can be acquired regardless of external lighting conditions with sub-centimeter-level spatial resolution. These properties make the TLS a potential remote sensing technique to study the circadian rhythm in trees. The main setback in detecting circadian movements with TLS is the requirement of stable environmental conditions, such as no wind and no precipitation, which present a significant technical challenge for long-term monitoring campaigns outdoors. So far, studies outside laboratory conditions are rare (Zlinszky et al., 2017; Puttonen et al., 2019). Here, we demonstrate with the second example that short-term structural phenomena are monitored with the measurement station as a future alternative for studying daily dynamics in trees. The main processing steps in the example are dataset selection, point cloud preprocessing, point cloud clustering, and cluster median coordinates comparison over time to monitor cluster movements.

First, a 30-h long dataset with no observed wind and without rainfall was selected by querying the SMEAR weather data. The point clouds in the dataset were acquired between April 30 at 12:00 AM (midnight) and May 01 at 5:00 A.M with a scan interval of 30 min. In total, the monitored time segment consisted of 60 scans. Temperature, precipitation, and prevalent wind conditions during scan times were interpolated from the 1-min interval weather measurements of the SMEAR station within 200 m from the test area. The average horizontal wind speed at 33.6 m height during the selected time segment was lower than 2 m/s between 30 April 12:00 AM (midnight). and April 30 5:00 P.M, after which it increased to 3 m/s for the rest of the time segment. The precipitation intensity was 0 mm/h, and the air temperature was between −1 and +5 degrees Celsius. Thus, we assume here that the measurement conditions was stable during the monitoring period and that any systematic structural changes between individual tree point clouds are mainly a result of intrinsic movements.

In the preprocessing step, the Silver birch used as sample data (Figure 3) was automatically segmented from the full point cloud dataset using range, scan angles and coordinates values as segmentation thresholds. A fine filtering was manually performed using Cloud Compare software to remove any isolated points and parts from neighboring trees remaining in the bounding box. Noise points were removed by looking up for points with no near neighbors, avoiding the influence of noises in the movement detection. The threshold condition used in the isolated point filter was to have at least 3 points within 5 cm 3D neighborhood. Furthermore, all tree point clouds were subsampled considering a minimum distance between points in the object space of 5 mm to decrease the time processing and to homogenize their point densities.

Different methods can be used to detect the circadian rhythm of trees. Puttonen et al. (2016, 2019) give a list of possible monitoring methods based on height percentiles, voxelization, clustering, skeletonization, and quantitative structure modeling. Here, we selected the point cloud clustering approach suggested by Puttonen et al. (2019) who used it to detect vertical movements in Norway maple branches overnight. The cluster monitoring approach was implemented in MATLAB 2017a. The algorithm can be explained in two steps. First, a user-selected (‘initial’) point cloud is segmented in clusters. Second, the initial cluster locations are projected over time by comparing the nearest neighbors between consecutive point clouds and labeling their points with the nearest cluster label.

In the initial clustering, random cluster seeds are first picked from the initial point cloud considering a minimum distance between existing cluster centers. The rest of the points are then assigned and labeled to the nearest cluster seed using a nearest neighbor search (minimum Euclidian distance). Then, the clustering is made more robust by removing small cluster with less than a minimum number of points and any points assigned to them are relabeled in the remaining clusters. In this example, a number of 100 points was set as a minimum accepted cluster size and the maximum initial cluster diameter was set at 0.15 m. After relabeling, median coordinates are calculated for each cluster. Once the initial clusters and cluster centers are defined, a nearest neighbor search is performed between the initial and the following consecutive scan point clouds. The median cluster coordinates are then used in determining the cluster movement direction and amplitude over time by comparing them with the initial cluster median coordinates. The circadian movements in different parts of the birch crown are determined from these cluster center coordinate differences compared with respect to the initial cluster center position.

**FIGURE 9:**
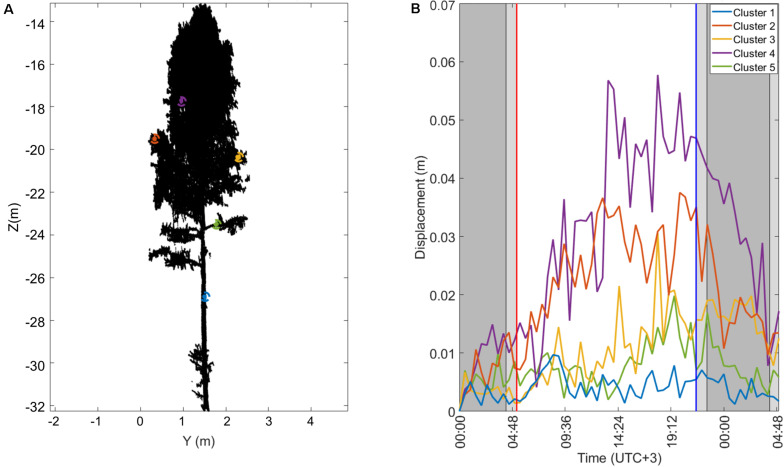
An example of a 30-h-long circadian rhythm monitoring period in a Silver birch tree between April 30 12 A.M and May 01, 2020 05:00 A.M using the TLS measurement station data. **(A)** Three segmented clusters selected from the stem and branches. **(B)** Selected cluster center displacements from the initial locations.

Figure 9 illustrates the results of the 30-h circadian rhythm monitoring in the Silver birch tree. Figure 9A illustrates three segmented clusters with one located on the tree stem (cluster 1) and two others on branches (clusters 2 and 3). Figure 9B presents the cluster median coordinate displacements with regard to their initial location on April 30 at 12 AM. The clusters start to move after sunrise, achieving a maximum displacement around 5 cm from their initial locations after noon. The sun rose 05:14 A.M on April 30 with the highest point at 1:20 P.M and set at 9:26 P.M. After sunset the clusters start to move back toward the initial position. This results show that the TLS measurement station provide point cloud time series with enough accuracy and resolution to monitor short-term dynamics, as previously presented by Puttonen et al. (2019).

## Conclusion and Outlooks

This work presents a technical description of the first permanent TLS measurement station and its data products developed and installed in Finland for long-term forest monitoring. This manuscript aims to enable the reproduction of similar systems for forest sciences and other study areas. The measurement station produces a high resolution spatial and temporal point cloud time series of a fixed forest scene. The point cloud time series provide detailed information on individual tree dynamics on a hectare level. The data enable a non-disruptive capture and storage of forest parameters and spatial-temporal dynamics in a natural environmental. The measurement station data can be compared with other sensor systems already present in the forest research station, such as weather parameters. We expect this to lead to new research and discussion about the potential that the measurement station can enable in studying both short- and long-term vegetation dynamics in a boreal forest. The measurement station is now operational and has been accumulating point cloud time series since March 2020. As a demonstration of the station performance, we presented here examples of both short- and long-term forest monitoring applications.

In the short-term, a 30-h monitoring period of a Silver birch tree was collected to determine its circadian rhythm dynamics. While several studies have already reported about the movement detection and quantification with TLS in individual trees, these studies are typically limited in their duration and are without accurate environmental reference measurements. This limits the possibilities to carry quantitative analysis of the driving factors behind plant movements and creates a bottleneck in studying the dynamics phenomena in natural forest plots. We believe that the measurement station will provide point cloud time series with a high enough spatial and temporal resolution to support the development of new methods for daily plant dynamics monitoring. The time-series data will directly support studies focusing in analyzing the possible mechanisms behind the branch movements, such as plant water balance or plant photoperiodism. Continuous time series of tree point clouds acquired from the test area under natural light conditions provide a unique dataset toward this goal. For instance, it is possible to observe the influence of lighting variation between sunset and sunrise on the internal clock of boreal trees in different seasons. At the same time, the wide areal coverage allows to compare both inter- and intra-species differences in circadian dynamics. However, clear challenges still exist to monitor circadian movements in trees on plot-level measurements that should be discussed. First, the environmental conditions, especially the wind speeds, during every scan acquisition, need to be carefully considered to guarantee the point cloud quality before doing quantitative analysis of plant movements. The wind speed can affect the natural targets and the tower stability. An alternative to minimize the influence of environmental conditions is to combine multi-sensors with different measurement principals in the measurement station. For instance, passive remote sensors with a near-instantaneous data acquisition (time-shot) can be added in future. Second, regular reference data collection of the tree geometry and physiology should be done in the test site to detect possible trends affecting data collection, but this is labor-intensive and time-consuming. And finally, the measurement station produces large quantities of raw point-cloud data whose efficient and fast handling requires the development of new processing techniques to make the full use of accumulating data.

In mid- to long-term monitoring, the measurement station can provide accurate information about weekly, monthly, and annual changes in the test forest. As a pioneer setup, the station will provide new insights about the accuracy, temporal resolution, and stability of TLS measurements and their repeatability in monitoring seasonal plants dynamics and in quantifying forest structure changes. We demonstrated that TLS time-series acquired with the measurement station can clearly capture the sprouting and growing of leaves during the spring growth season, thus enabling the accurate monitoring and timing of major phenological changes in the test site. However, regular field measurements are needed in future as a baseline to quantify all forest parameters in the test site that can be accurately monitored according to the tree-sensor distance. In the long term, detection of any drifts in the growth periods in boreal forests can offer important information that are related to climate change. If the measurement station concept proves useful, it can be extended in future to other similar monitoring sites and networks. This would open up new possibilities in monitoring the effects of climate change on a regional and global level.

The high spatial resolution and hectare-level coverage of the measurement station data also enable automatic estimation of traditional forest parameters, such as DBH, which are commonly used in an extensive range of forest applications. In order to make full use of the measurement station’s potential, more focused studies are needed to create fully automated data processing workflows to collect these parameters. This work can be extended to the development of a future TLS station for the seasonal monitoring of forest and agriculture areas. The monitoring of agriculture areas is out of the scope of this work. However, the technical description provided here can be used to extend this work for agricultural proposes (Crommelinck and Höfle, 2016; Hoffmeister et al., 2016; Guo et al., 2019), such as crop growth cycles monitoring and detection of height and biomass changes.

## Data Availability Statement

The original contributions generated for this study are included in the article/Supplementary Material, further inquiries can be directed to the corresponding author/s. Practical point cloud processing tools can be download at doi: 10.5281/zenodo.4309576.

## Author Contributions

MC, PL, YW, YC, HH, JH, and EP contributed directly in the development of the permanent TLS station. EP and JH contributed to the conception and physical design of the TLS station. HH developed the aluminum frame. The TLS station physical installation and field campaigns were performed by EP, HH, and MC. MC, EP, YW, YC, and PL contributed to the code implementation. MC and EP contributed to the data analysis and visualization methods presented in the manuscript, and wrote the manuscript’s first draft. All authors contributed to the manuscript revision, and read and approved the submitted version.

## Conflict of Interest

The authors declare that the research was conducted in the absence of any commercial or financial relationships that could be construed as a potential conflict of interest.

## Footnotes

1 https://www.atm.helsinki.fi/SMEAR/index.php/smear-ii

2 https://www.atm.helsinki.fi/SMEAR/index.php/smear-ii/measurements
