# Supplementary figures and images for: A Long-Term Terrestrial Laser Scanning Measurement Station to Continuously Monitor Structural and Phenological Dynamics of Boreal Forest Canopy
Source: Front Plant Sci. 2021 Jan 7;11:606752. doi: 10.3389/fpls.2020.606752 (PMC7817955; doi:10.3389/fpls.2020.606752)

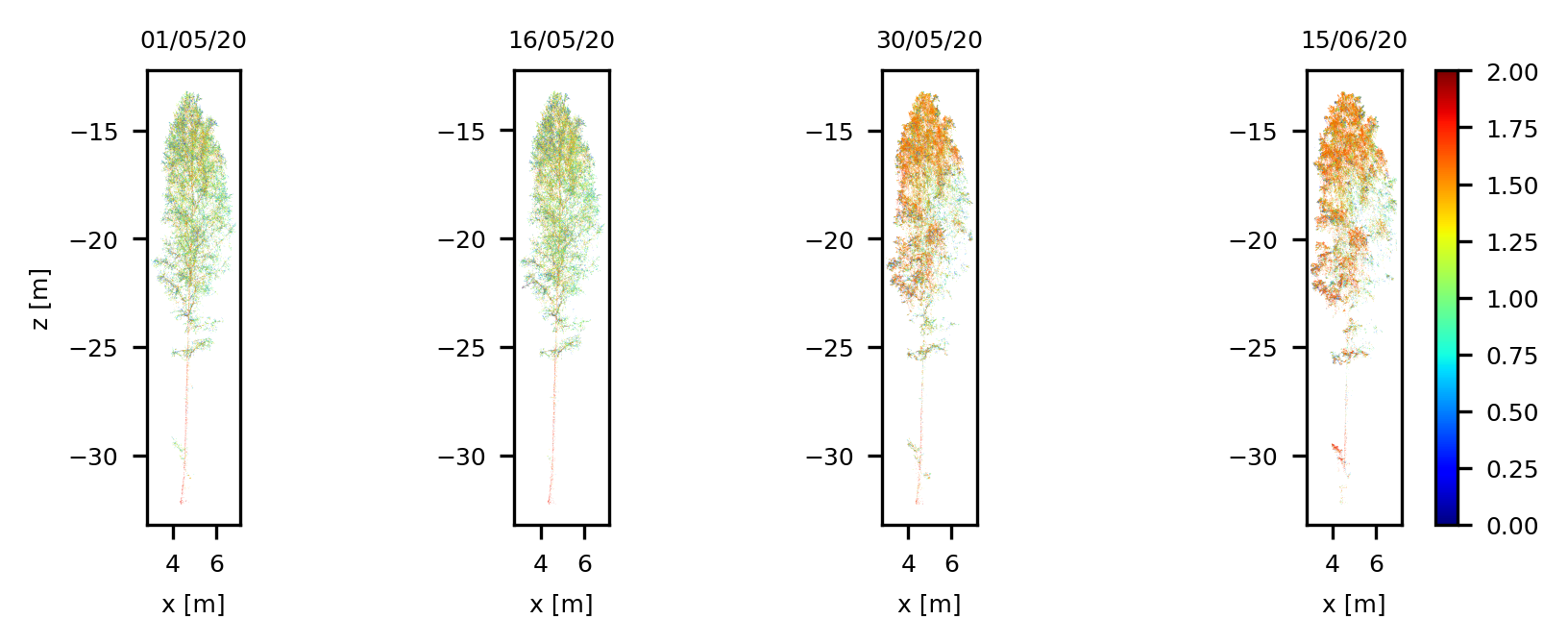

Supplement: Supplementary file 1 [file Data_Sheet_1.zip › FIGURE5/Figure5_ReflectanceColor02.png]
