# Supplementary material for: A Long-Term Terrestrial Laser Scanning Measurement Station to Continuously Monitor Structural and Phenological Dynamics of Boreal Forest Canopy
Source: Front Plant Sci. 2021 Jan 7;11:606752. doi: 10.3389/fpls.2020.606752 (PMC7817955; doi:10.3389/fpls.2020.606752)
Supplement: Supplementary file 3 [file Presentation_1.pdf]

Supplementary Material

1 Supplementary Data

This section describe the data available as supplementary material of the paper "A long-term terrestrial laser scanning measurement station to continuously monitor structural and phenological dynamics of boreal forest canopy".

1.1 Volumetric dynamics on a Silver birch crown

Data sheet 1 contains four point clouds of a Silver birch tree located at the Hyytiälä research forest (61°51´N, 24°17´E). The point clouds were acquired by the permanent TLS station between May and June in 2020, ranging from early-, middle-, late- spring, and early summer (May 1, 16, and 30 and June 15). File names are presented according to the data acquisition time in the format year (YY), month (MM), day (DD), hour (hh), minute (mm) and second (ss) (YYMMDD_hhmmss.laz). The Silver birch was located about 6 m away from the scan. This tree was 19 m tall and had a DBH of 173 mm. The Silver birch was delineated from the full point cloud dataset, using range, scan angles, and coordinates values as thresholds. The point clouds are in .laz format and they contain the 3D coordinates (X, Y, Z) of the points in a local reference system, point return number (scalar from 1 to 15), number of returns (scalar from 1 to 15), intensity (dB ranging from 0 to1), scan angles (theta and phi in degrees), reflectance (dB), return pulse deviation (measure of pulse shape distortion), range (m) and the internal scanner coordinates (row and column). The last five parameters were stored as user-defined extra bytes in the .laz content. Their values were computed using the 3D coordinates of the point cloud in a local reference system (X, Y, Z), in which the point cloud origin (100, 100, 100) is set to the laser scanner position. Each point cloud has approximately 68 MB. Additionally, a simple python script is provided, in which it is possible to reproduce Figure 5 presented in the paper. Therefore, it is possible to visualize the seasonal variation in the Silver birch crown during the spring 2020 growth season. The color scale used in the plot presents individual laser point reflectance in logarithmic scale (0–2.0). To run the code the user need to install python (https://www.python.org/ v. 3.7) and the python libraries numpy (https://numpy.org/), matplotlib (https://matplotlib.org/) and laspy (https://pypi.org/project/laspy/).

1.2 Changes on Silver birch stem diameter

Data sheet 2 contains sixteen point clouds from April to June, acquired by the permanent TLS station at April 4th, April 12th, April 19th, April 28th, May 4th, May 16th, May 23th and June 5th. Eight point clouds (YYMMDD_hhmmss_STEM.laz) are a short time series of a segment of the same Silver birch tree stem centered at 1.3 m height above the ground (± 60 cm around), ranging from 0.7 m to 1.9 m above the ground. The Silver birch tree is located at the Hyytiälä research forest (61°51´N, 24°17´E), which is 19 m tall with an approximate DBH of 173 mm. The others eight point clouds (YYMMDD_hhmmss_CYLINDER.laz) are referent to the cylinder surface (vertex) that was fitted to the segmented tree stem using the random sample consensus (RANSAC) method. The point clouds are in .laz format and they contain the 3D coordinates (X, Y, Z) of the points in a local reference system. A python plot script to reproduce Figure 8 is provided using the data described above. Therefore, it is

possible to visualize the seasonal variation in the Silver birch stem during the growth season. To run the code the user need to install python (https://www.python.org/ - version 3.7 was used) and the python libraries numpy (https://numpy.org/), matplotlib (https://matplotlib.org/) and laspy (https://pypi.org/project/laspy/).

2 TLS point cloud tools

Practical point cloud processing tools can be download at https://doi.org/10.5281/zenodo.4309576. The codes are available at: https://gitlab.com/eetun-tiimi/frontiers_606752. This section presents a briefly description of the scripts available as supplementary material, which included read and write las/.laz files in Python (nplas_tools) and MATLAB (MAT2LAS), convert .rxp to las/.laz files in C/C++ language (rxp2laz -) and Python tools to resample the point clouds and clipping individual trees from the complete point cloud scene. We would like to highlight that this tools were developed in the context of the permanent TLS station data (Frontiers manuscript no. 606752). Some adaptations can be required according to the users' application. The codes can be used as examples for other code developments. The authors welcome all comments for improving the toolbox and its documentation. The codes can be used according to MIT license principles. According to MIT license:

"Permission is hereby granted, free of charge, to any person obtaining a copy of this software and associated documentation files (the "software"), to deal in the software without restriction, including without limitation the rights to use, copy, modify, merge, publish, distribute, sublicense, and/or sell copies of the software, and to permit persons to whom the software is furnished to do so, subject to the following conditions: the software is provided "as is", without warranty of any kind, express or implied, including but not limited to the warranties of merchantability, fitness for a particular purpose and noninfringement. in no event shall the authors or copyright holders be liable for any claim, damages or other liability, whether in an action of contract, tort or otherwise, arising from, out of or in connection with the software or the use or other dealings in the software."

2.1 RXP2LAZ

The source code rxp2laz was developed in C/C++ language to converts .rxp Riegl format to LAS format (1.4) or - its compressed, but identical twin - the LAZ format, using RIVLIB and LASzip dynamic libraries (.dll). The source code can be compile at Windows or Linux platform. Instructions to user compilation can be found in the documentation at Gitlab folder. RIVLIB (http://www.riegl.com/index.php?id=224) and LASzip (https://laszip.org/) dynamic libraries are not provided, which must be obtained directly from the owners company with the appropriate copyright.

2.2 MAT2LAS

MAT2LAS is a MATLAB MEX code for using LAStools library functionalities to read and write las/.laz files to/from MATLAB structure, which consists of a reader function las2mat and a writer function mat2las. Instructions for compiling MAT2LAS in Linux or Windows can be found in the documentation at Gitlab folder. LASlib files are not provided. They can be obtained at https://github.com/LAStools/LAStools/tree/master/LASlib. The point-wise data are read and stored in MATLAB as a structure of vectors, enabling the development of MATLAB script for point cloud data processing, according to the user needs. After processing and point cloud manipulation the MATLAB structure can be exported by the user as las/laz file format (1.4).

2.3 NP2LAS

Similar to MAT2LAS, NP2LAS is a source code for using LAStools library functionalities to read and write las/.laz files to/from numpy arrays. The source code was developed in C/C++ language and generates as output a dynamic library (dll). The dll is converted to a reader and writer python functions using the python library ctypes (ReadLaz.py and WriteLaz.py). The source code can be compile at Windows or Linux platform. Instructions to user compilation and settings can be found in the documentation at Gitlab folder. LASlib files are not provided.
